# Associations of 24-hour movement behaviors with depressive symptoms in rural-dwelling older adults: a compositional data analysis

**DOI:** 10.1007/s40520-024-02827-2

**Published:** 2024-08-09

**Authors:** Tong Zhao, Rui Liu, Qi Han, Xiaolei Han, Juan Ren, Ming Mao, Jie Lu, Lin Cong, Yongxiang Wang, Shi Tang, Yifeng Du, Chengxuan Qiu

**Affiliations:** 1 Department of Neurology, Shandong Provincial Hospital, Shandong University, No. 324 Jingwuweiqi Road, Jinan, 250021 Shandong P.R. China; 2 Department of Neurology, Shandong Provincial Hospital Affiliated to Shandong First Medical University, Jinan, Shandong P.R. China; 3 Department of Ultrasound, Shandong Provincial Hospital Affiliated to Shandong First Medical University, Jinan, Shandong P.R. China; 4 Department of Neurology, Huashan Hospital, Fudan University, Shanghai, P.R. China; 5 Shandong Provincial Clinical Research Center for Neurological Diseases, Jinan, Shandong P.R. China; 6 Aging Research Center, Center for Alzheimer Research, Department of Neurobiology, Care Sciences and Society, Karolinska Institutet-Stockholm University, Stockholm, Sweden

**Keywords:** Sleep duration, Sedentary behavior, Physical activity, Depressive symptoms, Accelerometer, Population-based study

## Abstract

**Background:**

We aimed to explore the association of sleep duration with depressive symptoms among rural-dwelling older adults in China, and to estimate the impact of substituting sleep with sedentary behavior (SB) and physical activity (PA) on the association with depressive symptoms.

**Methods:**

This population-based cross-sectional study included 2001 rural-dwelling older adults (age ≥ 60 years, 59.2% female). Sleep duration was assessed using the Pittsburgh Sleep Quality Index. We used accelerometers to assess SB and PA, and the 15-item Geriatric Depression Scale to assess depressive symptoms. Data were analyzed using restricted cubic splines, compositional logistic regression, and isotemporal substitution models.

**Results:**

Restricted cubic spline curves showed a U-shaped association between daily sleep duration and the likelihood of depressive symptoms (*P*-nonlinear < 0.001). Among older adults with sleep duration < 7 h/day, reallocating 60 min/day spent on SB and PA to sleep were associated with multivariable-adjusted odds ratio (OR) of 0.81 (95% confidence interval [CI] = 0.78–0.84) and 0.79 (0.76–0.82), respectively, for depressive symptoms. Among older adults with sleep duration ≥ 7 h/day, reallocating 60 min/day spent in sleep to SB and PA, and reallocating 60 min/day spent on SB to PA were associated with multivariable-adjusted OR of 0.78 (0.74–0.84), 0.73 (0.69–0.78), and 0.94 (0.92–0.96), respectively, for depressive symptoms.

**Conclusions:**

Our study reveals a U-shaped association of sleep duration with depressive symptoms in rural older adults and further shows that replacing SB and PA with sleep or vice versa is associated with reduced likelihoods of depressive symptoms depending on sleep duration.

**Supplementary Information:**

The online version contains supplementary material available at 10.1007/s40520-024-02827-2.

## Introduction

Globally, depression is a major human blight that is responsible for more disability and death than any other health conditions [1]. As people age, the prevalence of depression increases [2], affecting 9.2% of older adults. Beyond personal suffering and family disruption, depression is associated with poor outcomes of various health conditions, disability, and even death of older adults [3].

Evidence from observational studies and randomized clinical trials has accumulated that healthy lifestyles such as healthy diet and regular physical activity (PA) are associated with a reduced risk of depression [4, 5]. In addition, several studies that examine the relationship between sleep duration and depression have yielded mixed results: some studies suggested cross-sectional and longitudinal associations of short sleep duration with depressive symptoms [6, 7], whereas others showed a cross-sectional association of both short and long sleep duration with depression in adults [8].

Of note, most of the previous studies have examined sleep in isolation, without considering the compositional and co-dependence nature of time use in sleep and other movement behaviors such as PA and sedentary behavior (SB), which might limit the interpretations of the study findings [9]. Given that a person’s time in a day is finite (i.e., 24 h), any increased time in sleep will displace the time spent in other types of behavior. The compositional data analysis (CoDA) is a novel statistical approach that could deal with multivariate data that forms part of a finite whole, such as time spent in sleep, SB, and PA adding up to the 24-hour a day [9, 10]. However, only one recent study used the CoDA method and found that replacing 30-minutes of sleep with moderate‑to‑vigorous PA was cross-sectionally related with a reduced likelihood of depressive symptoms among Caucasian older adults in Europe [11]. However, this study did not examine the potential nonlinear relationship between sleep duration and depressive symptoms, and thus, substituting sleep with other movement behaviors may have a different impact on the association with depressive symptoms depending on the daily sleep duration.

Furthermore, the association between sleep and depressive symptoms among Chinese rural older adults has not yet been well characterized. This is important because studies have suggested that rural-dwelling older adults are more likely to suffer from depression than urban older adults [12, 13]. The reasons are not fully understood, but could be due partly to limited education, low socioeconomic status, and life dissatisfaction in rural residents. In addition, having limited access to health care, being more likely to suffer from somatic disorders, and having insufficient knowledge of sleep hygiene among rural older adults might contribute to a higher prevalence of depressive symptoms [14, 15].

Therefore, in this population-based study, we sought to explore the association between sleep duration and depressive symptoms among rural-dwelling older adults in China and further to estimate the impact of substituting sleep with SB or PA on the association with depressive symptoms using CoDA method while taking into account their interdependent nature over 24 h.

## Methods

### Study design and participants

**Fig. 1:**
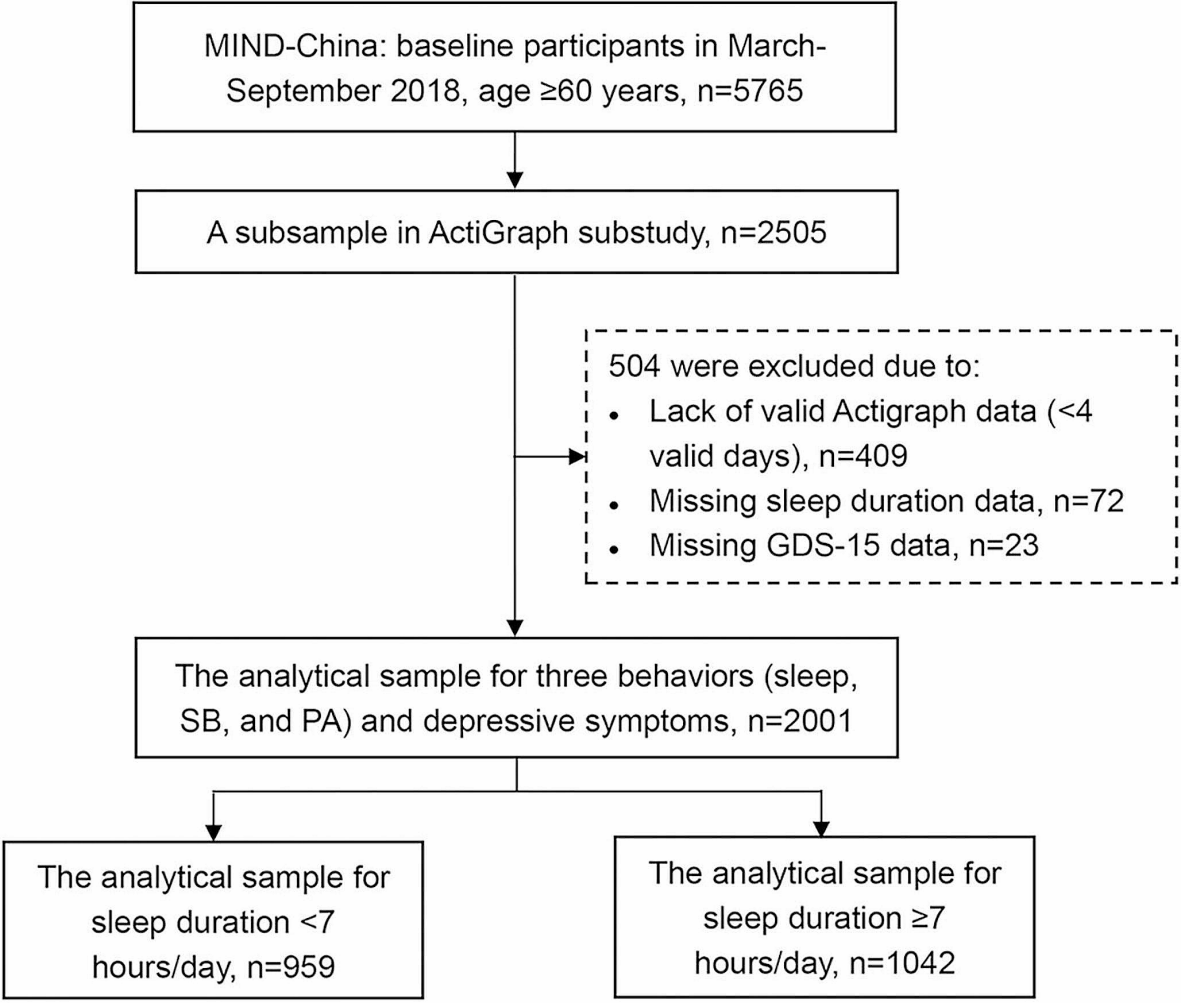
Flowchart of the study participants. Abbreviations: MIND-China, the Multimodal Interventions to Delay Dementia and Disability in Rural China; GDS-15, the 15-item Geriatric Depression Scale; SB, sedentary behavior; PA, physical activity

This population-based cross-sectional study used data from the baseline assessments of the Multimodal Interventions to Delay Dementia and Disability in Rural China (MIND-China) study, which was a participating project in the World-Wide FINGERS Network, a global network for risk reduction and prevention of dementia and cognitive disorders [16]. The comprehensive baseline assessments of MIND-China were previously reported in detail [17–19]. Briefly, MIND-China targeted people who were aged 60 years and older and living in the rural communities (52 villages) of Yanlou Town, Yanggu County in western Shandong Province. In March-September 2018, 5765 participants (74.9% of all eligible persons) underwent the baseline examination. In August 2018-December 2020, a subsample of 2505 participants in MIND-China underwent the ActiGraph examination. Of these, 504 participants were excluded due to insufficient wear time (< 4 valid days) of ActiGraph (*n* = 409) and missing data on sleep duration (*n* = 72) and depressive symptoms (*n* = 23), leaving 2001 participants for the current analyses. Figure 1 and Text S1 provide the flowchart and detailed description of the study participants.

The MIND-China study was approved by the Ethics Committee of Shandong Provincial Hospital. Research has been conducted in accordance with the Declaration of Helsinki. Written informed consent was obtained from the study participants or a proxy in the case of persons with severe cognitive impairment. MIND-China was registered in the Chinese Clinical Trial Registry (registration no.: ChiCTR1800017758).

### Measures

#### 24-hour movement behaviors

The 24-hour movement behaviors included sleep duration, SB, and PA. Sleep duration was assessed using the validated Chinese version of the Pittsburgh Sleep Quality Index (PSQI)[20– 22]. PA and SB were assessed during 7 consecutive days by ActiGraph wGT3X-BT triaxial accelerometer (ActiGraph, LLC, Pensacola, FL), as previously reported [18, 20]. Participants were asked to wear the accelerometer on their hips during waking hours, except while bathing or swimming activities [23]. Only participants who wore the device for ≥ 10 h/day in ≥ 4 valid days were included in the analyses [17, 24]. We defined SB as < 100 counts per minute, and PA ≥ 100 counts per minute [17].

#### Depressive symptoms

Depressive symptoms were measured using the 15-item Geriatric Depression Scale (GDS-15), which is a well-validated screening test for depression in older people [25]. The GDS-15 score ranges from 0 to 15, with a higher score indicating a higher likelihood of depression. The presence of depression symptoms was defined as the GDS-15 score ≥ 5 [25].

#### Covariates

Data on demographics, lifestyles, medical history, and use of medications were collected through the face-to-face interview, clinical examination, and laboratory tests [26, 27]. All medications were classified according to the Anatomical Therapeutic Chemical (ATC) classification system, as previously reported [27]. Education level was categorized as no formal schooling education, primary school, and middle school and above. Body mass index (BMI) was calculated as weight (kg) divided by height squared (m^2^). Smoking and alcohol drinking status were categorized as never, former, and current. Hypertension was defined as systolic pressure ≥ 140 mmHg or diastolic pressure ≥ 90 mmHg or current use of antihypertensive agents (ATC codes C02, C03, and C07-C09), diabetes as self-reported history of diabetes diagnosed by a physician or fasting blood glucose (FBG) ≥ 7.0 mmol/L or current use of antidiabetic agents (ATC code A10), and dyslipidemia as total cholesterol (TC) ≥ 6.22 mmol/L or triglyceride (TG) ≥ 2.27 mmol/L or low-density lipoprotein cholesterol (LDL-C) ≥ 4.14 mmol/L or high-density lipoprotein cholesterol (HDL-C) < 1.04 mmol/L or use of hypolipidemic agents (ATC code C10). Coronary heart disease was defined according to self-reported history or electrocardiogram examination, including angina pectoris, myocardial infarction, and coronary intervention. Stroke was ascertained according to self-reported history of stroke and neurological examination. Hypnotics included hypnotics and sedatives (ATC code N05C). ActiGraph wear season was categorized into spring, summer, autumn, and winter.

### Statistical analysis

Descriptive characteristics of study participants were presented with frequencies (%) for categorical variables and mean (standard deviation [SD]) for continuous variables. We compared the characteristics of the study participants by the presence of depressive symptoms using the chi-square test for categorical variables, t-test for continuous variables with normal distribution, and Kruskal-Wallis *H* test for those with skewed distribution.

Restricted cubic spline (RCS) analysis was performed to examine dose-response relationship between sleep duration and depressive symptoms and knots were placed at the 10th, 50th, and 90th percentiles. If RCS analysis suggested a linear relationship, sleep duration was analyzed as a continuous variable using multivariable general linear regression models. When the non-linear association was detected, we plotted the dose-response trajectories in RCS analysis. Then, we further analyzed the association of sleep duration with depressive symptoms stratified according to the inflection point.

CoDA was conducted followed the principles proposed by Chastin et al [10]. Based on the inflection point identified by the RCS analysis, we divided the study participant into those with a sleep duration < 7 h/day and a sleep duration ≥ 7 h/day. The associations of sleep duration (hours/day), SB, and PA with depressive symptoms among the two groups (i.e., sleep duration < 7 vs. ≥7 h) were examined using compositional logistic regression model. In brief, the daily time participants spent on each of the behaviors was considered a combination, which was normalized to the proportion of 1440 min (i.e., 100% of the available time in a given day). Time-use composition (sleep duration, PA, and SB) was expressed as a set of isometric log ratio (ilr) coordinates using the sequential binary partition process. The first coordinate represented the first part of the composition (either sleep, PA or SB) relative to all remaining behaviors. We reported the main results from two models: Model 1 was adjusted for age, sex, education, and ActiGraph wear season, and daily wearing time of ActiGraph for the analysis involving SB or PA; Model 2 was further adjusted for BMI, smoking, alcohol consumption, hypertension, diabetes, dyslipidemia, coronary heart disease, stroke, and hypnotics use. For the analysis of compositional isotemporal substitution, we presented the results only from Model 2. In this analysis, the expected changes in odds ratio (OR) of depressive symptoms associated with movement behaviors (e.g., sleep duration, SB, and PA) were estimated based on pairwise time-reallocation.

Because the use of hypnotics might confound the association between sleep duration and depressive symptoms, we conducted a sensitivity analysis by excluding the participants who used hypnotics to test the robustness of our findings.

We employed the R Statistical Software for Windows (version 4.2.0, R Foundation for Statistical Computing, Vienna, Austria) for all the analyses. The CoDA analysis was conducted using R packages “Compositions” version 2.0–4 and “robCompositions” version 2.3.1 for Windows (R Foundation for Statistical Computing, Vienna, Austria. URL https://www.R-project.org/). A two-tailed *P* < 0.05 was considered statistically significant.

## Results

### Characteristics of study participants

The mean age of the 2001 participants was 71.04 (SD, 4.79) years, 59.2% were female, 37.8% were illiterate (no formal schooling), and 191 (9.55%) were defined with depressive symptoms. Compared to participants without depressive symptoms, those with depressive symptoms were more likely to be female, to have coronary heart disease and stroke, and to use hypnotics, less educated, less likely to smoke and consume alcohol, and spent less time in sleep (*P* < 0.05). The two groups had no significant differences in age, BMI, hypertension, diabetes, dyslipidemia, wear seasons of ActiGraph, and daily SB and PA time (Table 1).

**Table 1:** Characteristics of study participants (*n* = 2001)

Characteristics	Total sample (*n* = 2001)	Depressive symptoms		
		No (*n* = 1810)	Yes (*n* = 191)	*P* value
Age, years	70.04 (4.79)	70.08 (4.86)	69.65 (4.13)	0.183
Female, n (%)	1184 (59.2)	1051 (58.1)	133 (69.6)	0.003
Educational level, n (%)				0.005
No school education	756 (37.8)	663 (36.6)	93 (48.7)	
Primary school	858 (42.9)	790 (43.6)	68 (35.6)	
Middle school and above	387 (19.3)	357 (19.7)	30 (15.7)	
Smoking, n (%)				0.011
Never	1335 (66.7)	1189 (65.7)	146 (76.4)	
Former	289 (14.4)	269 (14.9)	20 (10.5)	
Current	377 (18.8)	352 (19.4)	25 (13.1)	
Alcohol consumption, n (%)^a^				0.014
Never	1234 (61.7)	1096 (60.6)	138 (72.3)	
Former	153 (7.6)	143 (7.9)	10 (5.2)	
Current	598 (29.9)	555 (30.7)	43 (22.5)	
Body mass index, kg/m^2 a^	25.02 (3.61)	24.99 (3.60)	25.32 (3.73)	0.221
Hypertension, n (%)^a^	1364 (68.2)	1229 (67.9)	135 (70.7)	0.474
Diabetes, n (%)	309 (15.4)	271 (15.0)	38 (19.9)	0.092
Dyslipidemia, n (%)	481 (24.0)	425 (23.5)	56 (29.3)	0.088
Coronary heart disease, n (%)	441 (22.0)	381 (21.0)	60 (31.4)	0.001
Stroke, n (%)	282 (14.1)	240 (13.3)	42 (22.0)	0.001
Hypnotics use, n (%)	100 (5.0)	81 (4.5)	19 (9.9)	0.002
Wear seasons, n (%)				0.946
Spring	293 (14.6)	265 (14.6)	28 (14.7)	
Summer	692 (34.6)	625 (34.5)	67 (35.1)	
Autumn	810 (40.5)	731 (40.4)	79 (41.4)	
Winter	206 (10.3)	189 (10.4)	17 (8.9)	
Wear time, hours/day	14.12 (1.34)	14.14 (1.33)	13.99 (1.38)	0.136
Sedentary behavior, hours/day	8.24 (1.92)	8.25 (1.93)	8.18 (1.86)	0.652
Physical activity, hours/day	5.88 (2.03)	5.89 (2.02)	5.80 (2.09)	0.579
Sleep duration, hours/day	6.61 (1.70)	6.64 (1.64)	6.25 (2.14)	0.014

Data are mean (standard deviation), unless otherwise specified

^a^ Numbers of participants with missing values were 16 for alcohol consumption, 12 for body mass index, and 15 for hypertension. In the subsequent analyses, a dummy variable was created to represent a group of individuals with missing values, and continuous variable with missing values was replaced with a mean value

### Association between sleep duration and depressive symptoms

**Fig. 2:**
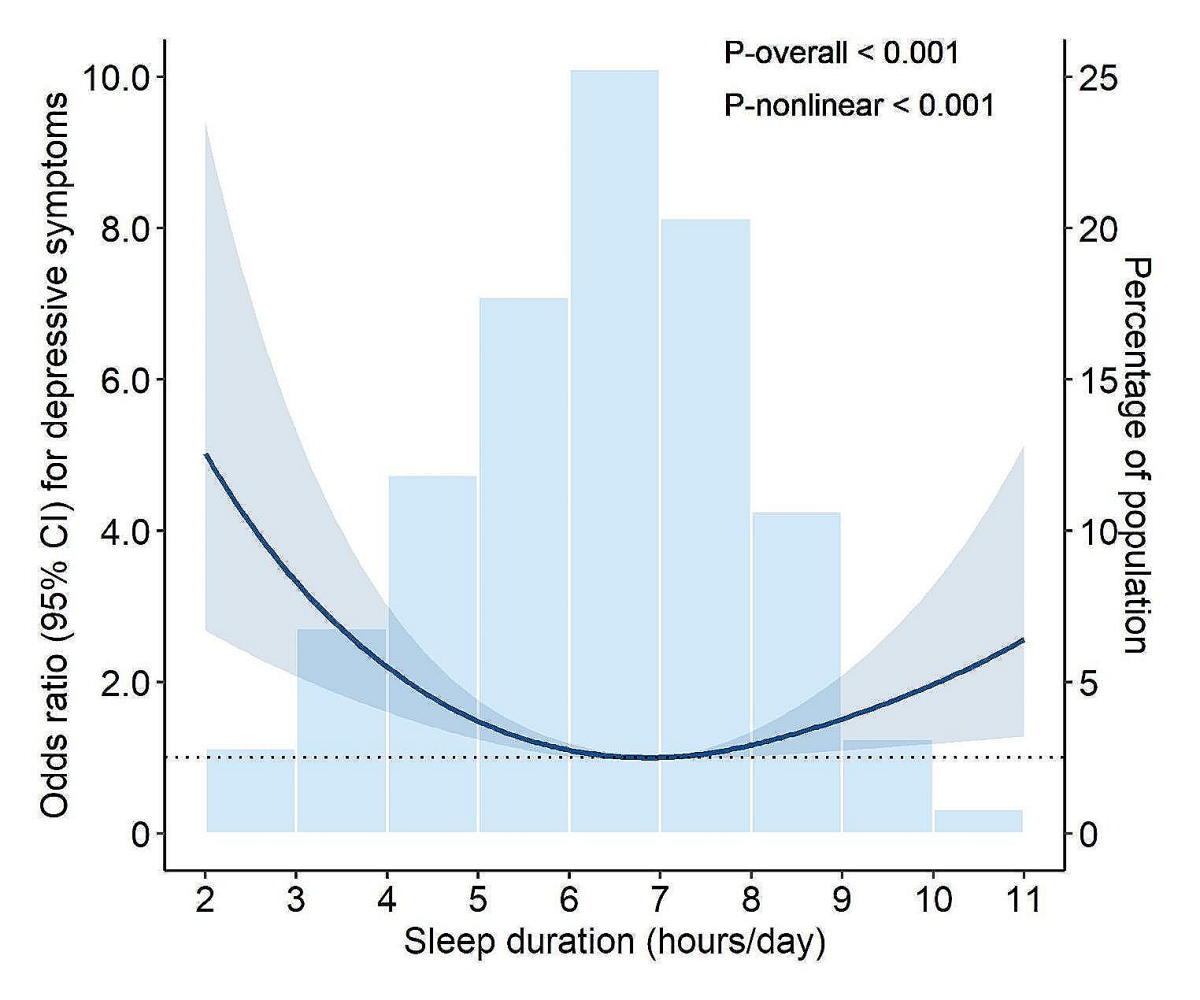
The multivariable-adjusted restricted cubic spline curves for the association between sleep duration and depressive symptoms (*n* = 2001). Solid line represented odds ratio of depressive symptoms, adjusting for age, sex, education, body mass index, smoking, alcohol consumption, hypertension, diabetes, dyslipidemia, stroke, coronary heart disease, and hypnotics use. The shaded areas represented the 95% confidence interval. The histogram represented the distribution of study participants. Abbreviation: CI, confidence interval

Adjusting for multiple potential confounders in Model 2, RCS curves showed a U-shaped association between sleep duration and the likelihood of depressive symptoms (*P*-nonlinear < 0.001), and the optimal sleep duration was found to be 7 h per day (Fig. 2). The logistic regression analysis showed that in participants with sleep duration < 7 h/day, longer sleep duration was associated with a decreased likelihood of depressive symptoms [adjusted odds ratio (OR) = 0.74; 95% confidence interval [CI] 0.63–0.88; *P* < 0.001]. Among participants with sleep duration ≥ 7 h/day, longer sleep duration was associated with an increased likelihood of depressive symptoms (adjusted OR = 1.31; 95% CI 1.05–1.64; *P* = 0.017). We did not detect a U-shaped relationship of SB or PA with depressive symptoms (data not shown).

### Time-use composition of movement behaviors

Time-use composition is presented as geometric means normalized to 24 h in different sleep duration groups (Supplementary Fig. 1). In the short sleep duration group (< 7 h/day), sleep occupied the smallest proportion (27%) and SB occupied the largest proportion (43%). In the long sleep duration group (≥ 7 h/day), SB and sleep occupied the proportions of 38% and 37%, respectively. PA occupied 30% in the short sleep duration group and 25% in the long sleep duration group.

### Associations between daily time-use composition of movement behaviors and depressive symptoms

In both short (< 7 h/day) and long (≥ 7 h/day) sleep duration groups, the daily time-use composition was significantly associated with depressive symptoms in both model 1 and model 2 (Table 2). Among older adults with sleep duration < 7 h/day, more time spent on PA and less time spent on sleep were significantly associated with an increased likelihood of depressive symptoms in both models. Longer sedentary time was associated with an increased likelihood of depressive symptoms in model 1, but the association did not reach statistical significance after further adjustment for covariates in model 2 (*P* = 0.053).

**Table 2:** Associations of movement behaviors with depressive symptoms by daily sleep duration (*n* = 2001)

Movement behaviors	Odds ratio (95% confidence interval), depressive symptoms	
	Model 1	Model 2
Sleep duration < 7 h/day (*n* = 959)		
Sedentary behavior, min/day	2.26 (1.20–4.28)^*^	1.93 (0.99–3.76)
Physical activity, min/day	1.87 (1.06–3.36)^*^	1.98 (1.11–3.61)^*^
Sleep duration, min/day	0.24 (0.11–0.50)^***^	0.26 (0.12–0.57)^***^
*P* for overall composition	< 0.001	0.005
Sleep duration ≥ 7 h/day (*n* = 1042)		
Sedentary behavior, min/day	0.38 (0.10–1.54)	0.47 (0.12–2.02)
Physical activity, min/day	0.23 (0.11–0.51)^***^	0.34 (0.15–0.80)^*^
Sleep duration, min/day	11.21 (1.61–73.89)^*^	6.28 (0.81–46.11)
*P* for overall composition	0.004	< 0.001

*P* for the overall composition was calculated using the likelihood ratio test. Time-use compositions were expressed as isometric log ratio (ilr) coordinates, and each result was from the initial ilr coordinates. The odds ratio corresponded to per one unit increase in ilr coordinates

Model 1 was adjusted for age, sex, education, ActiGraph wear season, and daily wear time for movement behaviors. Model 2 was additionally adjusted for body mass index, smoking, alcohol consumption, hypertension, diabetes, dyslipidemia, coronary heart disease, stroke, and hypnotics use. ^*^P < 0.05, ^**^P < 0.01, ^***^P < 0.001

Among older adults with sleep duration ≥ 7 h/day, less time spent on PA was significantly associated with an increased likelihood of depressive symptoms in both models. Longer sleep duration was associated with an increased likelihood of depressive symptoms in model 1, but the association did not reach statistical significance after further adjustment for covariates in model 2 (*P* = 0.074). There was no significant association between sedentary time and depressive symptoms (Table 2).

### Impact of replacing sleep with SB and PA on depressive symptoms

**Fig. 3:**
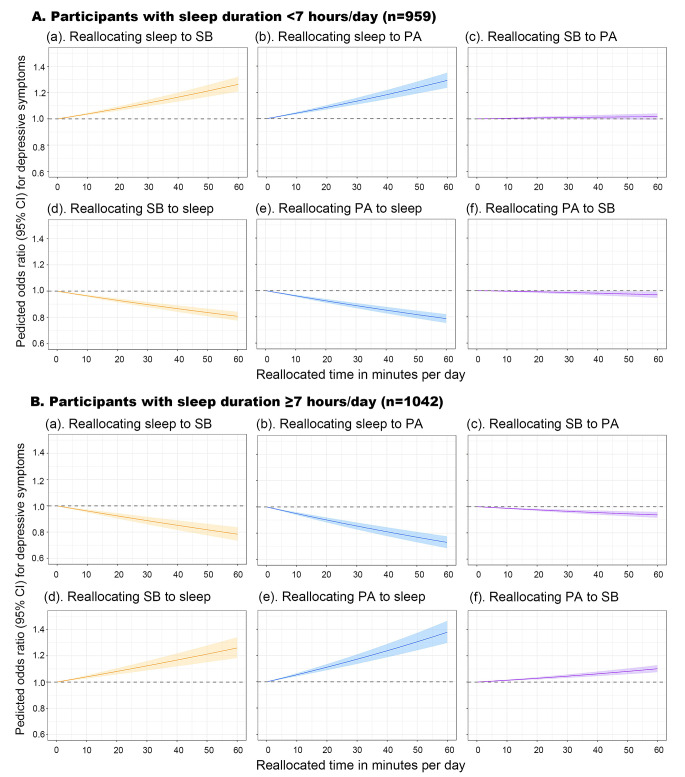
The predicted odds ratio and 95% confidence interval of depressive symptoms when reallocating a given amount of time among sleep, sedentary behavior, and physical activity while keeping the remaining components constant as compositional means by sleep duration (< 7 vs. ≥7 h/day) (*n* = 2001). Solid line represented odds ratio of depressive symptoms, adjusting for age, sex, education, body mass index, smoking, alcohol consumption, hypertension, diabetes, dyslipidemia, coronary heart disease, stroke, hypnotics use, ActiGraph wear season, and daily wear time. The shaded areas represented the 95% confidence interval. Abbreviations: OR, odds ratio; CI, confidence interval; SB, sedentary behavior; PA, physical activity

In compositional isotemporal substitution analyses, the associations of movement behaviors with depressive symptoms differed by sleep duration. For the short sleep duration group (< 7 h/day), reallocating 60 min per day spent on SB and PA to sleep were associated with multivariable-adjusted OR of 0.81 (95% CI 0.78–0.84) and 0.79 (0.76–0.82), respectively, for depressive symptoms. While among individuals with long sleep duration (≥ 7 h/day), reallocating 60 min per day spent in sleep to SB and PA and reallocating 60 min per day spent in SB to PA were associated with multivariable-adjusted OR of 0.78 (0.74–0.84), 0.73 (0.69–0.78), and 0.94 (0.92–0.96), respectively, for depressive symptoms (Fig. 3).

### Sensitivity analysis

We repeated the analyses by excluding 100 participants who used hypnotics, which yielded the results similar to those from the main analyses (Supplementary Tables 1 and Supplementary Figs. 2 & 3).

## Discussion

This population-based study of rural-dwelling Chinese older adults revealed U-shaped cross-sectional associations of sleep duration with depressive symptoms, with the optimal sleep duration being 7 h per day. The association held when using compositional methods to account for SB and PA over 24 h. Replacing SB or PA with sleep was associated with a reduced likelihood of depressive symptoms among older adults with sleep duration < 7 h/day, while replacing excessive sleep with SB or PA, and replacing SB with PA were associated with a reduced likelihood of depressive symptoms among older adults with sleep duration ≥ 7 h/day.

Previously, several population-based studies have explored the association between sleep duration and depressive symptoms among older adults, but the results are mixed. In the Northern Manhattan Study of racially/ethnically diverse population (69% Hispanic, 17% Black, and 14% White), compared to intermediate sleep durations (6–8 h/day), short sleep duration (< 6 h/day), but not long sleep duration (> 9 h/day), was cross-sectionally associated with depressive symptoms [6]. However, the differences in ethnicity, sociocultural background, socioeconomic status, and living habits between study populations may partly contribute to the inconsistent results. Moreover, a population-based cross-sectional study from Taiwan found that only long sleep duration (≥ 8 h/day) was associated with an increased likelihood of depression independent of various confounding factors [28]. However, our rural-dwelling older adults, who often engaged in heavy farmland labor work and had relatively limited access to healthcare and had limited personal health literacy, were more likely to suffer from somatic disorders and sleep disorders [19]. In addition, the multicenter cross-sectional study of community-dwelling older men from the USA found no association between objective total sleep time and depressive symptoms [29]. Differences in the study design, characteristics of study participants, assessment methods of sleep parameters, and control of potential confounders might partly contribute to the discrepant findings across studies. Our cross-sectional study found a U-shaped association between sleep duration and likelihoods of depressive symptoms, which needs to be interpreted in the context of Chinese culture and rural older adults with no or very limited education.

We also modeled the impact to provide more realistic estimates of replacing daily sleep time with SB and PA on the association with depressive symptoms. To the best of our knowledge, this is the first study to explore depressive symptoms in rural older adults using compositional data analysis approach. The cross-sectional study of older adults using data from NHANES in USA reported similar findings that replacing 60 min of SB with sleep was associated with a reduction of − 0.08 (95%CI − 0.10 to − 0.05) on the scores of depressive symptoms [30]. The compositional data analysis of community-dwelling older adults from the Seniors‑ENRICA‑2 study in Spain suggested that substituting 30 min of SB for sleep was associated with an increased likelihood of depression [11], which was contrary to the findings of the aforementioned NHANES study [30]. However, the aforementioned studies did not analyze the possible non-linear relationship between sleep duration and depressive symptoms. For older adults with long and short sleep duration, the impact of replacing sleep with SB or PA on its association with depressive symptoms appears to vary depending on the daily sleep duration. Indeed, we observed a U-shaped association between sleep duration and the likelihood of depressive symptoms (Fig. 2), therefore, we further explored the impact of replacing SB and PA on the association with depressive symptoms stratified by daily sleep duration (< 7 vs. ≥7 h/day). For older adults with a short sleep duration (< 7 h/day), replacing time spent on SB or PA with sleep was associated with a reduced likelihood of depressive symptoms (Fig. 3). This implies that in individuals with a relatively short sleep duration, compared to engaging time in SB or PA, spending more time on sleep seems more important than engaging SB or PA to counteract depressive symptoms. For older adults with a long sleep duration (≥ 7 h/day), excessive sleep was associated with an increased likelihood of depressive symptoms (Table 2). Furthermore, replacing excessive sleep with any other daily activities (SB or PA) was associated with a reduced likelihood of depressive symptoms, of which, replacing sleep time with PA showed the most substantial impact. In addition, replacing time spent on SB with PA was also associated with a slightly reduced likelihood of depressive symptoms (Fig. 3). This suggests that for individuals already with long sleep time, reducing sleep time and increasing other movement behaviors time, especially PA time, may reduce depressive symptoms.

Several potential mechanisms may explain the associations between sleep duration and depressive symptoms. Short sleep could increase chronic inflammation [31, 32], and was associated with inflammatory cytokines such as C-reactive protein and interleukin-6 [33]. Short sleep duration could also lead to daytime physical exhaustion [34] and daytime sleepiness, which may alter circadian rhythms or cause endocrine hormone changes [35, 36], and further result in depressive symptoms. Long sleep duration has been also associated with proinflammatory biomarkers (e.g., interleukin-6 and C-reactive protein) [37, 38], which may be the pathways linking long sleep duration to depressive symptoms. Furthermore, people with a long sleep duration tend to have less time for PA, which will elicit changes in neuroplasticity, inflammation, oxidative stress, the endocrine system, self-esteem, social support, and self-efficacy [39].

The major strength of this study was the large-scale population-based study of rural-dwelling older adults who had relatively low socioeconomic status and received no or limited education. We also used the CoDA approach which considered the interdependence of time use in different movement behaviors when exploring the association of sleep duration with depressive symptoms and estimated the effects of their replacement between 24-hour movement behaviors. In addition, the objectively-measured SB and PA parameters can minimize recall bias and improve the reliability of the study findings.

Nonetheless, our study has limitations. First, we could not infer any causal relationship through this cross-sectional study. Second, there was a time gap between assessments of baseline characteristics (March-September 2018) and collection of ActiGraph data (August 2018-December 2020), which should be taken into account when interpreting the results. Third, sleep duration and depressive symptoms were assessed through self-reported information, although PSQI and GDS-15 have been widely used in the population-based studies of older adults. Fourth, we did not have data on the percentage of people who were still engaging in work, including farm work in agriculture, while having regular work could affect time spent on SB and PA. Finally, the study participants were recruited from only one rural region in western Shandong Province and may not be representative of the entire rural populations in China, which should be kept in mind when generalizing the study findings to other populations.

## Conclusion

Our study shows that both short and long sleep duration are associated with an increased likelihood of depressive symptoms in rural Chinese older adults, even when taking into account the intrinsically compositional nature of the time spent in 24-hour movement behaviors. We further revealed that among older adults with sleep duration < 7 h/day, reallocating time spent on PA and SB to sleep was associated with lower likelihoods of depressive symptoms, and that among older adults with sleep duration ≥ 7 h/day, reallocating time spent in sleep to PA and SB was associated with lower likelihoods of depressive symptoms. These results contribute to our understanding of the relationships of different movement behaviors (i.e., sleep, SB, and PA) with depressive symptoms in rural older adults. Further long-term prospective cohort studies using objective sleep measures (e.g., polysomnography and actigraphy) will help to clarify the temporal causal relationships between sleep duration and depression as well as the impact of changing the proportion of daily time spent on different movement behaviors on the risk of depression, which is crucial for the development of preventive interventions.

### Electronic supplementary material

Below is the link to the electronic supplementary material.

Supplementary Material 1

## Data Availability

No datasets were generated or analysed during the current study.

## Declarations

### Data statement

The datasets used and/or analyzed during the current study are available from the corresponding author upon reasonable request.
