# Supplementary material for: Associations of 24-hour movement behaviors with depressive symptoms in rural-dwelling older adults: a compositional data analysis
Source: Aging Clin Exp Res. 2024 Aug 9;36(1):165. doi: 10.1007/s40520-024-02827-2 (PMC11315720; doi:10.1007/s40520-024-02827-2)
Supplement: Supplementary file 1 — Supplementary Material 1 [file 40520_2024_2827_MOESM1_ESM.docx]

**Supplementary materials**

**Associations of** **24-hour movement behaviors with depressive symptoms in rural-dwelling older adults: a compositional data analysis**

Zhao T, et al.

**Contents**

**Supplementary Text S1.** Methods.

**Supplementary Table 1.** Associations of movement behaviors with depressive symptoms in participants who were free of hypnotics use by daily sleep duration (n=1901)

**Supplementary Figure 1.** Estimated distribution of movement behaviors over 24 hours

**Supplementary Figure 2.** The multivariable-adjusted restricted cubic spline curves for the association between sleep duration and depressive symptoms in participants who were free of hypnotics use (n=1901)

**Supplementary Figure 3.** The predicted odds ratio and 95% confidence interval of depressive symptoms when reallocating a given amount of time among sleep, sedentary behavior, and physical activity while keeping the remaining components constant as compositional means by sleep duration (<7 vs. ≥7 hours/day) (n=1901)

**Supplementary Text S1.** Methods.

**Study design and participants**

Participants in the MIND-China ActiGraph substudy were selected following the procedure described below. Following the baseline assessments in MIND-China in March-September 2018, a total of 4276 persons from 32 villages were randomly selected from all the 52 villages in Yanlou Town to participate in the ActiGraph substudy. Of these, after explaining the procedure of the ActiGraph examination, 1408 persons were too busy to participate, 117 died before examination, 23 had limited physical function and were not able to participate, and 212 lost the contact, leaving 2516 (58.8%) participants who agreed to wear the ActiGraph accelerometer. Of these, we further excluded 11 participants due to loss of ActiGraph data or technical problems or returned unworn. Of the 2505 participants who had the ActiGraph data, 504 were excluded due to insufficient wear time (<4 valid days) of ActiGraph (n=409) and missing data on sleep duration (n=72) or depressive symptoms (n=23), leaving 2001 participants for the current analyses.

**Supplementary Table 1.** Associations of movement behaviors with depressive symptoms in participants who were free of hypnotics use by daily sleep duration (n=1901)

| Movement behaviors | Odds ratio (95% confidence interval), depressive symptoms | |
| --- | --- | --- |
|  | Model 1 | Model 2 |
| Sleep duration <7 hours/day (n=959) |  |  |
| Sedentary behavior, min/day | 2.46 (1.26-4.87)^**^ | 2.12 (1.06-4.30)^*^ |
| Physical activity, min/day | 1.73 (0.95-3.23) | 1.84 (1.00-3.48) |
| Sleep, min/day | 0.23 (0.11-0.52)^***^ | 0.26 (0.11-0.58)^**^ |
| *P* for overall composition | 0.003 | 0.026 |
| Sleep duration ≥7 hours/day (n=1042) |  |  |
| Sedentary behavior, min/day | 0.34 (0.09-1.42) | 0.40 (0.09-1.79) |
| Physical activity, min/day | 0.24 (0.11-0.55)^***^ | 0.35 (0.15-0.85)^*^ |
| Sleep, min/day | 12.41 (1.65-87.53)^*^ | 7.16 (0.86-56.28) |
| *P* for overall composition | 0.017 | <0.001 |

*P* for the overall composition was calculated using the likelihood ratio test. Time-use compositions were expressed as isometric log ratio (ilr) coordinates, and each result was from the initial ilr coordinates. The odds ratio corresponded to per one unit increase in ilr coordinates.

Model 1 was adjusted for age, sex, education, ActiGraph wear season, and daily wear time for movement behaviors. Model 2 was additionally adjusted for body mass index, smoking, alcohol consumption, hypertension, diabetes, dyslipidemia, coronary heart disease, and stroke. ^*^*P*<0.05, ^**^*P*<0.01, ^***^*P*<0.001.


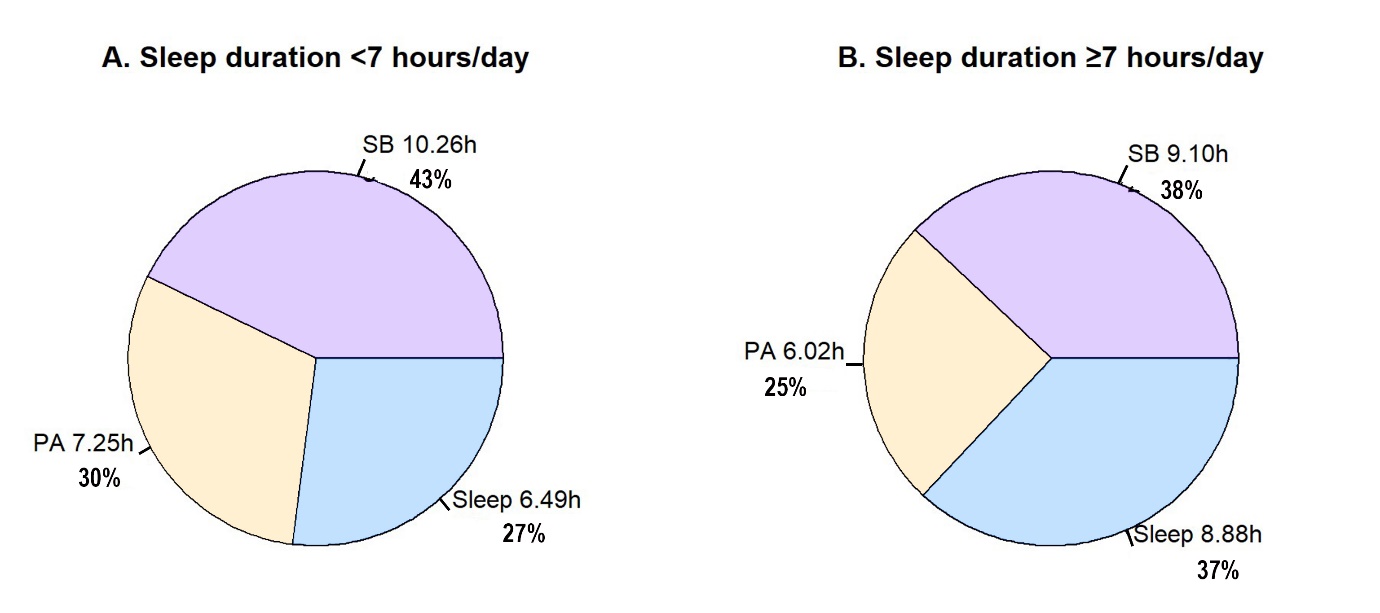


**Supplementary Figure 1.** Estimated distribution of movement behaviors over 24 hours.

Time-use composition is presented as geometric means normalized to 24 hours in different sleep duration groups (<7 vs. ≥7 hours/day).

Abbreviations: SB, sedentary behavior; PA, physical activity.


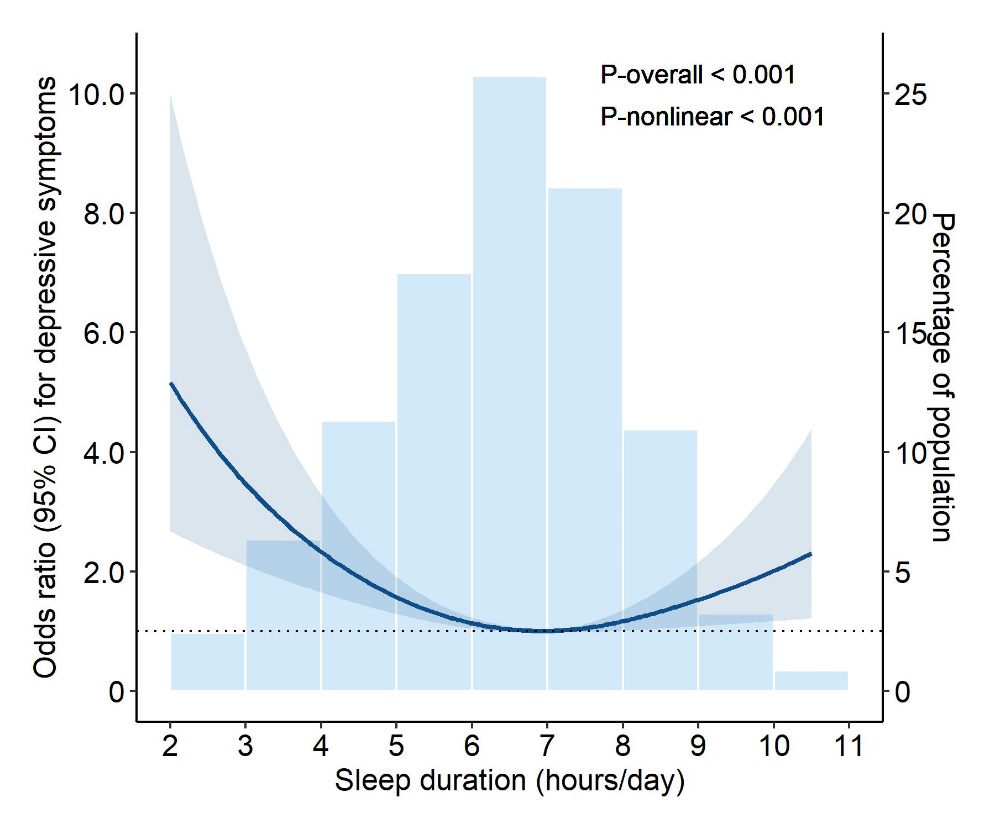


**Supplementary Figure 2.** The multivariable-adjusted restricted cubic spline curves for the association between sleep duration and depressive symptoms in participants who were free of hypnotics use (n=1901)

Solid line represented odds ratio of depressive symptoms, adjusting for age, sex, education, body mass index, smoking, alcohol consumption, hypertension, diabetes, dyslipidemia, stroke, and coronary heart disease. The shaded areas represented the 95% confidence interval. The histogram represented the distribution of study participants.

Abbreviation: CI, confidence interval.


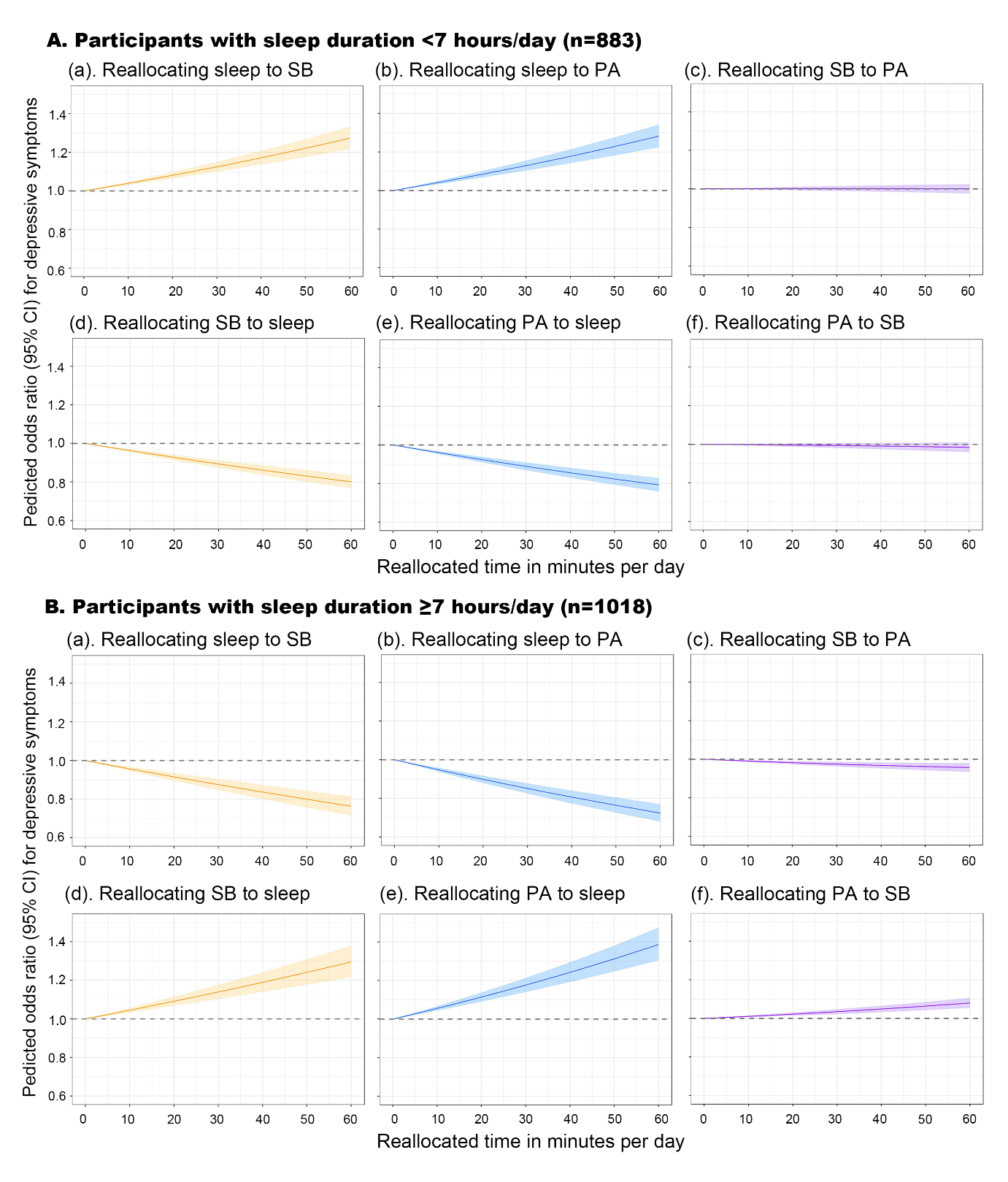


**Supplementary Figure 3.** The predicted odds ratio and 95% confidence interval of depressive symptoms when reallocating a given amount of time among sleep, sedentary behavior, and physical activity while keeping the remaining components constant as compositional means by sleep duration (<7 vs. ≥7 hours/day) (n=1901)

Solid line represented odds ratio of depressive symptoms, adjusting for age, sex, education, body mass index, smoking, alcohol consumption, hypertension, diabetes, dyslipidemia, coronary heart disease, stroke, ActiGraph wear season, and daily wear time. The shaded areas represented the 95% confidence interval.

Abbreviations: OR, odds ratio; CI, confidence interval; SB, sedentary behavior; PA, physical activity.
